# Peripheral blood transcriptomes and a single-cell atlas suggest a monocyte-associated immune recovery-failure axis in STEMI

**DOI:** 10.1371/journal.pone.0358983

**Published:** 2026-09-29

**Authors:** Qi Cheng, Qunxing Li, Delu Yin

**Affiliations:** The Affiliated Lianyungang Hospital of Xuzhou Medical University/ The First People’s Hospital of Lianyungang, Jiangsu, Lianyungang, China; IRCCS San Raffaele Pisana, ITALY

## Abstract

**Background:**

Patients with ST-segment elevation myocardial infarction (STEMI) continue to show substantial inflammatory heterogeneity after reperfusion therapy, and recovery trajectories may differ markedly across patients. Compared with traditional diagnostic models, characterizing the dynamic immune trajectory from acute inflammation toward recovery may better identify programs associated with impaired resolution. This study aimed to construct an immune recovery-failure framework that is transferable across cohorts and anchored by single-cell data.

**Methods:**

We integrated six public GEO peripheral-blood bulk transcriptome cohorts and one public single-cell RNA-seq dataset. An acute-to-recovery immune axis was constructed from directionally concordant longitudinal and cross-sectional contrasts, and a recovery-failure phenotype was defined in the discovery longitudinal cohort from paired transcriptomic features. An 18-gene monocyte-associated module was then derived using bulk recovery-failure associations, monocyte specificity, monocyte-state differences, and pseudotime information. External cohorts were used to evaluate temporal transfer and baseline-to-follow-up associations rather than definitive clinical prediction, and the single-cell dataset was used for donor-aware cell-type anchoring and exploratory state-level interpretation.

**Results:**

Among 81 STEMI patients who met the longitudinal definition criteria, unsupervised stratification classified 35 as recovery-failure patients and 46 as recovery-sufficient patients. We constructed an 18-gene monocyte-associated recovery-failure module. The module distinguished the two transcriptomically defined groups in the discovery cohort and declined from the acute phase toward recovery in external longitudinal cohorts. Baseline module burden was associated with later composition-adjusted inflammatory burden, but this was interpreted as a baseline-to-follow-up association rather than clinical event prediction. At the single-cell level, donor-aggregated lesion-associated shifts were largest in monocytes, and the module decreased along the acute-high-to-recovery-biased monocyte pseudotime direction. In 1,000 matched random monocyte-gene modules, the observed module showed a higher aggregate recovery statistic than nearly all random signatures.

**Conclusions:**

We propose a STEMI immune recovery-failure framework derived from public peripheral-blood transcriptomes, evaluated across independent longitudinal datasets, and linked to monocyte state variation in a public single-cell context. The framework is does not evidence of a causal mechanism or a validated hard-endpoint prediction model; rather, it provides a reproducible transcriptomic description of impaired inflammatory resolution that should be tested prospectively and experimentally.

## Introduction

Acute coronary syndrome (ACS) remains a major contributor to cardiovascular death and disability worldwide, with STEMI representing the most severe and prototypical phenotype of acute ischemic necrosis [1–3]. In recent years, the understanding of ACS has shifted from a simple focus on the degree of coronary stenosis toward a dynamic framework involving plaque rupture, plaque erosion, inflammatory activation, and host repair capacity [4–9]. Although reperfusion therapy has substantially improved early survival, a subset of patients still exhibits persistent inflammation, delayed repair, and a tendency toward adverse remodeling after revascularization [10–19]. Therefore, moving beyond whether acute injury is present to whether inflammation is effectively resolved and recovery fails represents a clinically more interpretable direction for patient stratification.

Prior peripheral-blood transcriptomic studies have described AMI-associated expression patterns and outcome-associated RNA profiles, but many analyses remain cross-sectional, single-cohort, or endpoint-driven [20–22]. In contrast, several public cohorts contain longitudinal blood transcriptomes spanning acute onset to later recovery, and GSE269269 provides a single-cell PBMC dataset from AMI patients with plaque rupture or non-rupture lesions. The key challenge is not to build yet another signature, but to define an immune state axis that has longitudinal directionality, can be carried by a lesion-associated single-cell context, and remains transferable across platforms.

Monocytes and monocyte-derived macrophages have long been considered key cellular populations linking acute necrosis, inflammatory clearance, and tissue repair [12–19]. After myocardial infarction, monocytes are not static; they undergo state transitions from acute inflammation-driven programs toward recovery and remodeling-biased states [13–19]. Human atherosclerosis and ACS-related single-cell studies have also shown that myeloid populations are highly plastic lesion-associated immune compartments [23–26]. We therefore hypothesized that a transcriptomic phenotype of incomplete inflammatory resolution, if recovered from paired peripheral-blood data and evaluated in a single-cell AMI context, would be most consistent with monocyte state variation rather than by the superficial phenomenon of increased cell abundance alone.

Based on this rationale, we used public data to construct a monocyte-centered immune recovery-failure framework in STEMI. Our objectives were to define an acute-to-recovery immune axis and a recovery-failure phenotype in paired bulk transcriptomes; construct a monocyte-associated recovery-failure module while explicitly separating internal evaluation from external temporal transfer; evaluate baseline-to-follow-up associations in independent longitudinal cohorts; perform donor-aware cell-type anchoring and pseudotime analysis in lesion-associated single-cell data; and assess robustness using matched random gene sets and multi-metric comparisons.

## Methods

### Study design and overall analytical workflow

This study was a secondary analysis of public transcriptomic datasets. The workflow was organized as follows: 1) retrieval and preprocessing of bulk and single-cell GEO data; 2)construction of cell-type marker sets from GSE269269 for later composition adjustment; 3) definition of a bulk acute-to-recovery immune axis using GSE59867, GSE61144, and GSE60993; 4) construction of a continuous recovery-failure index (RFI) and binary recovery-failure phenotype in GSE59867; 5) construction of an 18-gene monocyte-associated recovery-failure module in the discovery cohort; 6) evaluation of temporal transfer and baseline-to-follow-up associations in GSE62646 and GSE123342; 7) donor-aware single-cell anchoring, monocyte-state pseudobulk analysis, ligand-receptor scoring, transcription-factor activity inference, and pseudotime analysis in GSE269269; and 8) robustness assessment using multi-metric benchmarks and matched random modules. Analyses derived from GSE59867 were considered discovery or internal evaluation; analyses in GSE62646 and GSE123342 were considered external temporal-transfer analyses; endpoint analyses in GSE59867 and GSE48060 were treated as exploratory boundary analyses.

### Definitions of derived scores and analytical independence

For a gene set S, the signature score in each sample or cell was defined as the mean row-standardized expression of genes in S that were present in that dataset. The acute-to-recovery axis was defined as mean_z(acute-program genes) – mean_z(recovery-program genes). The monocyte-adjusted axis was the standardized residual from axis_score ~ Monocyte_score within each dataset, and the composition-adjusted axis was the standardized residual from axis_score ~ Monocyte_score + lymphoid_score + Platelet_score. The continuous RFI was defined in GSE59867 as the sum of four standardized patient-level features: baseline composition-adjusted axis, 6-month composition-adjusted axis, longitudinal area under the composition-adjusted axis curve, and change from baseline to month 6. The binary recovery-failure phenotype was assigned by k-means clustering of these four standardized features and was therefore an unsupervised transcriptomic phenotype rather than a clinical endpoint label.

The recovery-failure module score was defined as mean_z(positive-arm genes) – mean_z(negative-arm genes). Module residual adjustment used the same composition covariates as the adjusted axis. The monocyte-state transfer score was defined as mean_z(acute-state genes) – mean_z(recovery-state genes), followed by composition residualization when used in bulk cohorts. The temporal recovery statistic used in robustness benchmarking was the negative paired t statistic for follow-up minus acute/baseline values, so larger positive values indicate stronger decline from acute phase toward recovery. Baseline-to-follow-up association models used an early-time score as the predictor and later composition-adjusted axis as the outcome; these models were not interpreted as independent clinical prediction of hard outcomes.

### Public datasets and assignment of analytical roles

We included six peripheral-blood bulk transcriptome cohorts and one PBMC single-cell RNA-seq cohort (Table 1; S1 Fig). GSE59867 was used as the discovery backbone for the acute-to-recovery axis, RFI, and binary recovery-failure phenotype; GSE61144 and; GSE60993 contributed to axis construction and directionality checking; GSE62646 and GSE123342 were used for external temporal-transfer and baseline-to-follow-up association analyses; GSE48060 was retained only as an exploratory endpoint-validation cohort; and GSE269269 was used for cell-type marker derivation, donor-aware single-cell anchoring, monocyte-state analysis, and pseudotime analysis. Primary source publications were identified from the GEO Series records and cited for each dataset: GSE59867 (Maciejak et al. [27]), GSE60993 and GSE61144 (Park et al. [28]), GSE62646 (Kiliszek et al. [29]), GSE123342 (Vanhaverbeke et al. [20] and Veltman et al. [30]), GSE48060 (Suresh et al. [21]), and GSE269269 (Qian et al. [31]).

**Table 1:** Public datasets included in this study.

Dataset	Data type	Main samples and time structure
GSE59867	Peripheral-blood mononuclear-cell bulk transcriptome microarray	Stable coronary artery disease, 46 cases; STEMI admission, 111 cases; discharge, 101 cases; 1 month, 95 cases; 6 months, 83 cases
GSE61144	Peripheral-blood bulk transcriptome microarray	Controls, 10 cases; STEMI pre-intervention, 7 cases; day 7 after intervention, 7 cases
GSE60993	Peripheral-blood bulk transcriptome microarray	Controls, 7 cases; unstable angina, 9 cases; non-ST-segment elevation myocardial infarction, 10 cases; STEMI, 7 cases
GSE62646	Peripheral-blood mononuclear-cell bulk transcriptome microarray	Controls, 14 cases; STEMI admission, 28 cases; discharge, 28 cases; 6 months, 28 cases
GSE123342	Whole-blood bulk transcriptome microarray	Controls, 22 cases; acute myocardial infarction day 0, 65 cases; day 30, 64 cases; 1 year, 37 cases (technical replicates removed)
GSE48060	Peripheral-blood bulk transcriptome microarray	Controls, 21 cases; acute myocardial infarction, 31 cases, including 5 recurrent events
GSE269269	Peripheral-blood mononuclear-cell single-cell RNA sequencing	10 patients (5 rupture, 5 non-rupture); 93,325 cells after quality control

### GSE269269 single-cell preprocessing and annotation

For GSE269269, raw 10x-style matrix, feature, and barcode files were downloaded from GEO and read for each sample. Seurat objects were created with min.cells = 3 and min.features = 200. Cells were retained if nFeature_RNA was between 200 and 7,000 and mitochondrial percentage was <= 25%. Data were log-normalized with Seurat NormalizeData, 3,000 variable features were selected by the vst method, and scaled data were used for PCA with 30 components. Harmony integration by sample_id was used when the package was available; otherwise PCA was used directly. Neighbors and UMAP were constructed from dimensions 1:20, and clustering used resolution 0.5. The reproducible analysis retained 93,325 cells.

### Preprocessing of bulk transcriptome cohorts and gene-level alignment

Bulk GEO Series Matrix files were retrieved with GEOquery or from the NCBI GEO download endpoints. We used the expression matrices deposited with the original studies and did not apply cross-study batch correction, because each dataset was analyzed within cohort and transferred by gene-set scoring rather than by pooling expression values. Probe identifiers were mapped to gene symbols using GEO platform annotations or Bioconductor annotation packages when required. When multiple probes mapped to the same gene, the mean expression across all corresponding probes was used. Genes with ambiguous or Genes with ambiguous or unstable transfer behavior were removed before scoring; these included LOC and LINC noncoding transcript labels, MIR and small RNA labels, ribosomal protein genes (RPL/RPS), mitochondrial genes (MT-), and annotation-like probe symbols beginning with common platform-specific prefixes. Signature scores were calculated after row-wise standardization within each dataset so that cross-platform differences in absolute expression scale did not directly determine the score.

### Construction of the acute-to-recovery immune axis

The acute-to-recovery immune axis was discovered using three complementary contrasts. GSE59867 compared paired differential analysis was performed between STEMI admission and 6-month PBMC samples, GSE61144 comparedpaired STEMI pre-PCI and day-7 peripheral-blood samples, a cross-sectional comparison between controls and GSE60993 compared STEMI with controls. Paired contrasts used limma with duplicateCorrelation to account for repeated measurements from the same patient. For each gene, t statistics from the three contrasts were standardized and integrated as 1.25 x z(t_GSE59867) + 0.90 x z(t_GSE61144) + 0.90 x z(t_GSE60993), giving slightly greater weight to the longitudinal discovery cohort while retaining external directionality. Genes were required to be stable and present in at least two cohorts. The 40 acute-program genes were selected from directionally consistent candidate tiers prioritizing GSE59867 FDR, cross-cohort directional support, integrated t score and 40 recovery-program genes, and mean absolute log fold change. The axis score was then calculated as the acute-program signature score minus the recovery-program signature score in each bulk cohort.

### Single-cell-guided composition adjustment of bulk transcriptomes

Because bulk blood transcriptomes are vulnerable to shifts in major immune-cell composition, we used GSE269269 single-cell data to construct cell-type marker sets were constructed from GSE269269 before bulk composition adjustment. For monocytes, T cells, NK cells, B cells, and platelets, cell-type specificity was calculated as the average expression in the target cell type minus the maximum average expression in the other profiled cell types. Marker genes required specificity > 0.3, detection in at least three bulk cohorts, and stable gene symbols; the top 30 markers per cell type were used to estimate bulk cell-signal scores. The lymphoid score was the mean of T-cell, NK-cell, and B-cell scores. Within each bulk dataset, monocyte-adjusted and composition-adjusted axes were generated as standardized residuals from linear models containing Monocyte_score alone or Monocyte_score + lymphoid_score + Platelet_score, respectively.

### Recovery-failure index and stratification

The recovery-failure phenotype was defined only in GSE59867. Eligible We included STEMI patients required admission and 6-month samples at least three longitudinal time points; and finite baseline, 6-month, and cumulative composition-adjusted axis values. Four patient-level features were extracted: baseline composition-adjusted axis, persistent 6-month composition-adjusted axis, longitudinal area under the composition-adjusted axis curve using trapezoidal integration over admission, discharge, 1 month, and 6 months when available, and change from baseline to month 6. All four features were standardized; missing standardized values after eligibility filtering were set to zero only if required for matrix construction. K-means clustering was used two centers, 200 random starts, and seed 20260415. The cluster with the higher sum of mean baseline, 6-month, cumulative, and clearance-burden features was labeled recovery-failure. The continuous RFI was the row sum of the four these standardized features. Longitudinal group differenceswere evaluated with linear mixed models using patient-specific random intercepts.

### Construction of the recovery-failure module

The recovery-failure module was constructed in GSE59867 and should be interpreted as discovery-cohort feature construction. At admission and 6 months: limma models tested gene-level associations with the continuous RFI; and between-group differences between recovery-failure and recovery-sufficient patients. Candidate genes also required monocyte specificity from GSE269269 (specificity > 0.5 and average monocyte expression > 0.5) and stable gene symbols. Pseudotime support was defined from the complete monocyte gene-pseudotime correlation table after orienting pseudotime from acute-high to recovery-biased monocytes. Positive candidates had positive admission RFI association and were prioritized by pmax(admission RFI logFC, 0) x specificity x log1p(average monocyte expression); negative candidates used the analogous negative association score. The final redundancy-pruned module contained eight positive-arm genes (CXCR2, SMAP2, CSF3R, FKBP5, DUSP1, IL1R2, C5AR1, and CEBPB) and ten negative-arm genes (S100A8, FTL, MNDA, CST3, LST1, TYROBP, CD14, AIF1, S100A10, and HLA-DRA). The module score was calculated as the positive-arm score minus the negative-arm score and was composition-adjusted by residualizing against Monocyte_score, lymphoid score, and Platelet_score within each bulk transcriptome cohort.

### External longitudinal transfer and baseline-to-follow-up association analysis

We evaluated the longitudinal direction of module change and its baseline-to-follow-up association in GSE62646 and GSE123342. For paired time points (GSE62646: admission versus 6 months; GSE123342: day 0 versus day 30), linear mixed models with patient-specific random intercepts were used when paired identifiers were available. The GSE123342 day 0 versus 1-year contrast was analyzed with a linear model because complete pairing was not available for all samples after technical replicate removal. Baseline-to-follow-up association models used the earlier module score as the predictor and the later composition-adjusted axis as the outcome in standardized linear regression. These analyses quantify temporal transfer and inflammatory-burden association, not independent prediction of clinical events.

### GSE269269 single-cell preprocessing and annotation

For GSE269269, raw 10x-style matrix, feature, and barcode files were downloaded from GEO and read for each sample. Seurat objects were created with min.cells = 3 and min.features = 200 [32, 33]. Cells were retained if nFeature_RNA was between 200 and 7,000 and mitochondrial percentage was <= 25%. Data were log-normalized with Seurat NormalizeData, 3,000 variable features were selected by the vst method, and scaled data were used for PCA with 30 components. Harmony integration by sample_id was used when the package was available; otherwise PCA was used directly [34]. Neighbors and UMAP were constructed from dimensions 1:20, and clustering used resolution 0.5. The reproducible analysis retained 93,325 cells.

### Preprocessing of bulk transcriptome cohorts and gene-level alignment

We Bulk GEO Series Matrix files were retrieved with GEOquery or from the NCBI GEO download endpoints [35, 36]. We used the expression matrices deposited with the original studies and did not apply cross-study batch correction, because each dataset was analyzed within cohort and transferred by gene-set scoring rather than by pooling expression values. Probe identifiers were mapped to gene symbols using GEO platform annotations or Bioconductor annotation packages when required. When multiple probes mapped to the same gene, the mean expression across probes was used. Genes with ambiguous or unstable transfer behavior were removed before scoring; these included LOC and LINC noncoding transcript labels, MIR and small RNA labels, ribosomal protein genes (RPL/RPS), mitochondrial genes (MT-), and annotation-like probe symbols beginning with common platform-specific prefixes. Signature scores were calculated after row-wise standardization within each dataset so that cross-platform differences in absolute expression scale did not directly determine the score.

### Construction of the acute-to-recovery immune axis

The acute-to-recovery immune axis was discovered using three complementary contrasts. GSE59867 compared paired STEMI admission and 6-month PBMC samples, GSE61144 compared paired STEMI pre-PCI and day-7 peripheral-blood samples, and GSE60993 compared STEMI with controls. Paired contrasts used limma with duplicateCorrelation to account for repeated measurements from the same patient [37]. For each gene, t statistics from the three contrasts were standardized and integrated as 1.25 x z(t_GSE59867) + 0.90 x z(t_GSE61144) + 0.90 x z(t_GSE60993), giving slightly greater weight to the longitudinal discovery cohort while retaining external directionality. Genes were required to be stable and present in at least two cohorts. The 40 acute-program genes and 40 recovery-program genes were selected from directionally consistent candidate tiers prioritizing GSE59867 FDR, cross-cohort directional support, integrated t score, and mean absolute log fold change. The axis score was then calculated as the acute-program signature score minus the recovery-program signature score in each bulk cohort.

### Single-cell-guided composition adjustment of bulk transcriptomes

Because bulk blood transcriptomes are vulnerable to shifts in major immune-cell composition, cell-type marker sets were constructed from GSE269269 before bulk composition adjustment. For monocytes, T cells, NK cells, B cells, and platelets, cell-type specificity was calculated as the average expression in the target cell type minus the maximum average expression in the other profiled cell types. Marker genes required specificity > 0.3, detection in at least three bulk cohorts, and stable gene symbols; the top 30 markers per cell type were used to estimate bulk cell-signal scores. The lymphoid score was the mean of T-cell, NK-cell, and B-cell scores. Within each bulk dataset, monocyte-adjusted and composition-adjusted axes were generated as standardized residuals from linear models containing Monocyte_score alone or Monocyte_score + lymphoid_score + Platelet_score, respectively (S2 Fig).

### Recovery-failure index and stratification

The recovery-failure phenotype was defined only in GSE59867. Eligible STEMI patients required admission and 6-month samples, at least three longitudinal time points, and finite baseline, 6-month, and cumulative composition-adjusted axis values. Four patient-level features were extracted: baseline composition-adjusted axis, persistent 6-month composition-adjusted axis, longitudinal area under the composition-adjusted axis curve using trapezoidal integration over admission, discharge, 1 month, and 6 months when available, and change from baseline to month 6. All four features were standardized; missing standardized values after eligibility filtering were set to zero only if required for matrix construction. K-means clustering used two centers, 200 random starts, and seed 20260415. The cluster with the higher sum of mean baseline, 6-month, cumulative, and clearance-burden features was labeled recovery-failure. The continuous RFI was the row sum of the four standardized features. Longitudinal group differences were evaluated with linear mixed models using patient-specific random intercepts.

### Construction of the recovery-failure module

The recovery-failure module was constructed in GSE59867 and should be interpreted as discovery-cohort feature construction. At admission and 6 months, limma models tested gene-level associations with the continuous RFI and between-group differences between recovery-failure and recovery-sufficient patients. Candidate genes also required monocyte specificity from GSE269269 (specificity > 0.5 and average monocyte expression > 0.5) and stable gene symbols. Pseudotime support was defined from the complete monocyte gene-pseudotime correlation table after orienting pseudotime from acute-high to recovery-biased monocytes. Positive candidates had positive admission RFI association and were prioritized by pmax(admission RFI logFC, 0) x specificity x log1p(average monocyte expression); negative candidates used the analogous negative association score. The final redundancy-pruned module contained eight positive-arm genes (CXCR2, SMAP2, CSF3R, FKBP5, DUSP1, IL1R2, C5AR1, and CEBPB) and ten negative-arm genes (S100A8, FTL, MNDA, CST3, LST1, TYROBP, CD14, AIF1, S100A10, and HLA-DRA). The module score was calculated as the positive-arm score minus the negative-arm score and was composition-adjusted by residualizing against Monocyte_score, lymphoid_score, and Platelet_score within each bulk cohort.

### External longitudinal transfer and baseline-to-follow-up association analysis

We evaluated the longitudinal direction of module change and its baseline-to-follow-up association in GSE62646 and GSE123342. For paired time points (GSE62646 admission versus 6 months; GSE123342 day 0 versus day 30), linear mixed models with patient-specific random intercepts were used when paired identifiers were available. The GSE123342 day 0 versus 1-year contrast was analyzed with a linear model because complete pairing was not available for all samples after technical replicate removal. Baseline-to-follow-up association models used the earlier module score as the predictor and the later composition-adjusted axis as the outcome in standardized linear regression. These analyses quantify temporal transfer and inflammatory-burden association, not independent prediction of clinical events.

### Single-cell module anchoring and within-cell-type standardization

The recovery-failure module was projected to each cell in GSE269269 by calculating positive-arm, negative-arm, and difference scores. Cell-level values were used for visualization and distributional description only. For group comparisons, cells were aggregated by donor, lesion group, and cell type or monocyte state, and Wilcoxon rank-sum tests were applied to donor-level summaries. For Figure 4C, the effect size was defined as the rupture minus non-rupture mean donor-level within-cell-type standardized module score; comparisons used 5 rupture and 5 non-rupture donors. To separate cell abundance from within-cell-type state shifts, module scores were also standardized within each cell type before rupture versus non-rupture comparisons. Thus, the independent unit for lesion-group inference was the patient, not the individual cell.

### Monocyte state, communication, and pseudotime analyses

Within monocytes, eligible clusters with at least 300 cells were ranked by the original acute-to-recovery axis score. The two highest-axis clusters were labeled acute-high and the two lowest-axis clusters were labeled recovery-biased. For differential expression between these two states, counts were aggregated into pseudobulk profiles by patient and monocyte state; pseudobulk samples required at least 25 cells. edgeR DGEList objects were normalized with calcNormFactors, transformed with voom, and modeled with limma using duplicateCorrelation with patient as the blocking factor [37,38]. Hallmark pathway enrichment used MSigDB Hallmark gene sets and fgsea [39, 40]. Ligand-receptor analysis used an OmniPath-derived expanded database filtered to common resources including CellChatDB, CellPhoneDB, CellTalkDB, ICELLNET, connectomeDB2020, and CellCall [41]. Sender and receiver expression were aggregated by patient, lesion group, and state with a minimum of 20 cells; ligand and receptor detection required expression in at least 10% of sender and receiver cells, and interaction scores were calculated as log1p(sender average expression x receiver average expression).

Monocyte pseudotime analysis was oriented from the acute-high state toward the recovery-biased state to match the acute-to-recovery interpretation. Slingshot was run on the first 10 monocyte PCA dimensions using Seurat clusters as labels, the highest-axis acute-high cluster as the start cluster, and the lowest-axis recovery-biased cluster as the end cluster [42]. Pseudotime values were scaled to 0–1, and cells with finite pseudotime were divided into ten equal-size pseudotime bins. Gene-pseudotime trends were summarized by Spearman correlation and expression change between the last and first bins. Transcription-factor activity was inferred on bin-level average expression using the dorothea human regulon restricted to confidence levels A-C and the decoupleR univariate linear model method [43, 44]. All pseudotime, ligand-receptor, and transcription-factor analyses were interpreted as observational state-level support rather than perturbational evidence of mechanism.

### Robustness analysis

We assessed robustness from three perspectives. First, temporal recovery ability was compared across metrics using the recovery statistic, defined as the negative paired t statistic for follow-up minus acute/baseline values; larger positive values therefore indicate stronger decline from acute phase toward recovery. Second, baseline-to-follow-up association was compared using standardized regression coefficients from models in which each baseline metric was associated with the subsequent composition-adjusted axis. Third, matched random-module analysis sampled positive and negative arms with the same arm sizes as the observed module from monocyte-centered candidate pools that excluded the observed module genes and were present in at least three bulk cohorts. Each random signature was scored, composition-adjusted, and evaluated with the same temporal recovery statistic. We increased the random sampling to 1,000 iterations. The empirical P value was calculated as (1 + number of random statistics >= the observed statistic)/ (1 + number of random iterations).

### Statistical analysis

Continuous variables are presented as means, standard deviations, or standard errors as appropriate. Unless otherwise stated, regression predictors and outcomes used for cross-metric comparisons were standardized before model fitting. Paired longitudinal bulk contrasts used linear mixed models with patient-specific random intercepts when repeated measurements were available; limma paired differential-expression analyses used duplicateCorrelation with patient as a blocking factor. Unpaired bulk contrasts used linear models with the response and predictors specified in the corresponding Methods subsection. Single-cell group inference used donor-level summaries or pseudobulk models rather than treating cells as independent biological replicates. Cell-level correlations and UMAP distributions were treated as descriptive. Wilcoxon rank-sum tests were two-sided and applied to donor-level summaries unless explicitly described otherwise. The Figure 4C donor-level cell-type comparisons are reported as nominal P values and descriptive effect-size summaries because of the small donor number, not as FDR-controlled discovery tests. Correlations used Spearman’s method. P values from gene-level differential expression, pathway enrichment, and ligand-receptor interaction summaries were adjusted using the Benjamini-Hochberg false-discovery-rate procedure. Confidence intervals were calculated as estimate + /- 1.96 x standard error for regression summaries. All analyses were performed in R 4.5.2.

## Results

1. Public Peripheral-Blood Cohorts Support a Quantifiable STEMI Recovery-Failure Phenotype

First, we constructed a cross-cohort directionally consistent acute-to-recovery immune axis in GSE59867, GSE61144, and GSE60993, and extended it to all bulk transcriptome cohorts. In GSE59867, STEMI patients were then stratified by unsupervised clustering using four composition-adjusted features: baseline axis burden, persistent 6-month burden, longitudinal cumulative burden, and clearance magnitude. In total, 81 patients met the phenotype-definition criteria; 35 were classified as recovery-failure patients and 46 as recovery-sufficient patients. The mean baseline composition-adjusted axis was 1.293 in the recovery-failure group and −0.160 in the recovery-sufficient group. At 6 months, both groups declined, but the recovery-failure group retained a higher residual burden (0.379 versus −0.775). These findings indicate that interpatient differences are reflected not only in the magnitude of acute-phase burden, but also in whether inflammatory burden is truly cleared during recovery (Fig 1).

**Fig 1:**
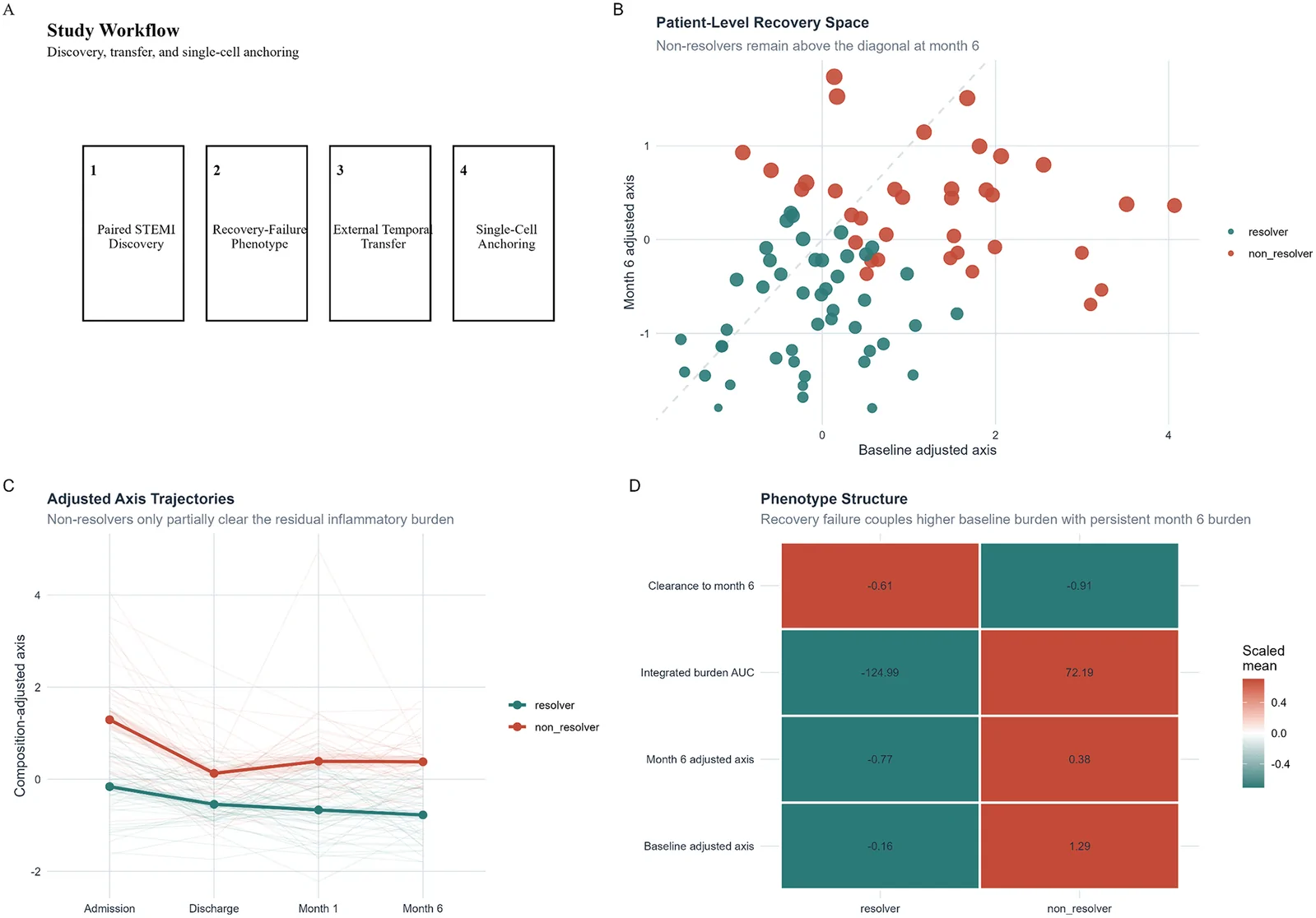
Construction of the recovery-failure phenotype. A, Overall study workflow. B, Patient-level scatter plot of the baseline composition-adjusted axis versus the 6-month composition-adjusted axis; point size indicates the continuous recovery-failure index. C, Longitudinal trajectories of the composition-adjusted axis in recovery-sufficient and recovery-failure groups. D, Heatmap of the four core features defining the recovery-failure phenotype.

The longitudinal trajectories further supported this stratification. Both individual trajectories and group means showed that recovery-sufficient patients gradually moved into a negative or near-baseline recovery range after hospitalization, whereas recovery-failure patients also declined but did not fully clear the burden and still retained substantial residual axis burden at 6 months. Because this phenotype was derived from each patient’s longitudinal structure rather than from a single time-point threshold, it more closely approximates the clinical concept of whether recovery has failed than a conventional cross-sectional high-versus-low grouping.

2. An 18-Gene Monocyte-Centered Recovery-Failure Module Is Constructed and Stably Supported in the Discovery Cohort

To translate the patient-level recovery-failure phenotype into a more transferable molecular program, we performed gene-level association analyses at admission and 6 months in GSE59867 and integrated these results with single-cell monocyte specificity, monocyte-state differential genes, and pseudotime-dynamic genes. This yielded an 18-gene module (S3 Fig). The module consisted of eight positive genes (CXCR2, SMAP2, CSF3R, FKBP5, DUSP1, IL1R2, C5AR1, and CEBPB) and ten negative genes (S100A8, FTL, MNDA, CST3, LST1, TYROBP, CD14, AIF1, S100A10, and HLA-DRA) (Table 2).

**Table 2:** Composition of the monocyte-centered recovery-failure module.

Module arm	Gene composition	Summary of evidence sources
Positive arm (8 genes)	CXCR2, SMAP2, CSF3R, FKBP5, DUSP1, IL1R2, C5AR1, CEBPB	Positively associated with the admission recovery-failure index, highly monocyte-specific, and partially supported by the acute-high state or early trajectory end
Negative arm (10 genes)	S100A8, FTL, MNDA, CST3, LST1, TYROBP, CD14, AIF1, S100A10, HLA-DRA	Negatively associated with the admission recovery-failure index, highly monocyte-specific, and partially supported by the recovery-biased state or late trajectory end

After composition adjustment, the recovery-failure module showed consistent internal associations in the discovery cohort. At admission, module burden was higher in the recovery-failure group than in the recovery-sufficient group (β = 1.108, 95% CI 0.705 to 1.511, P = 7.30e-07). At 6 months, this difference persisted (β = 0.769, 95% CI 0.386 to 1.152, P = 1.79e-04). When recovery failure was treated as a continuous spectrum, the module was positively associated with the continuous RFI at admission (β = 0.164, P = 2.73e-04) and at 6 months (β = 0.233, P = 3.14e-10). These internal results indicate that the module varied continuously along the transcriptomically defined recovery-failure gradient, but they do not constitute independent validation because both the phenotype and module were constructed from GSE59867 (Fig 2).

**Fig 2:**
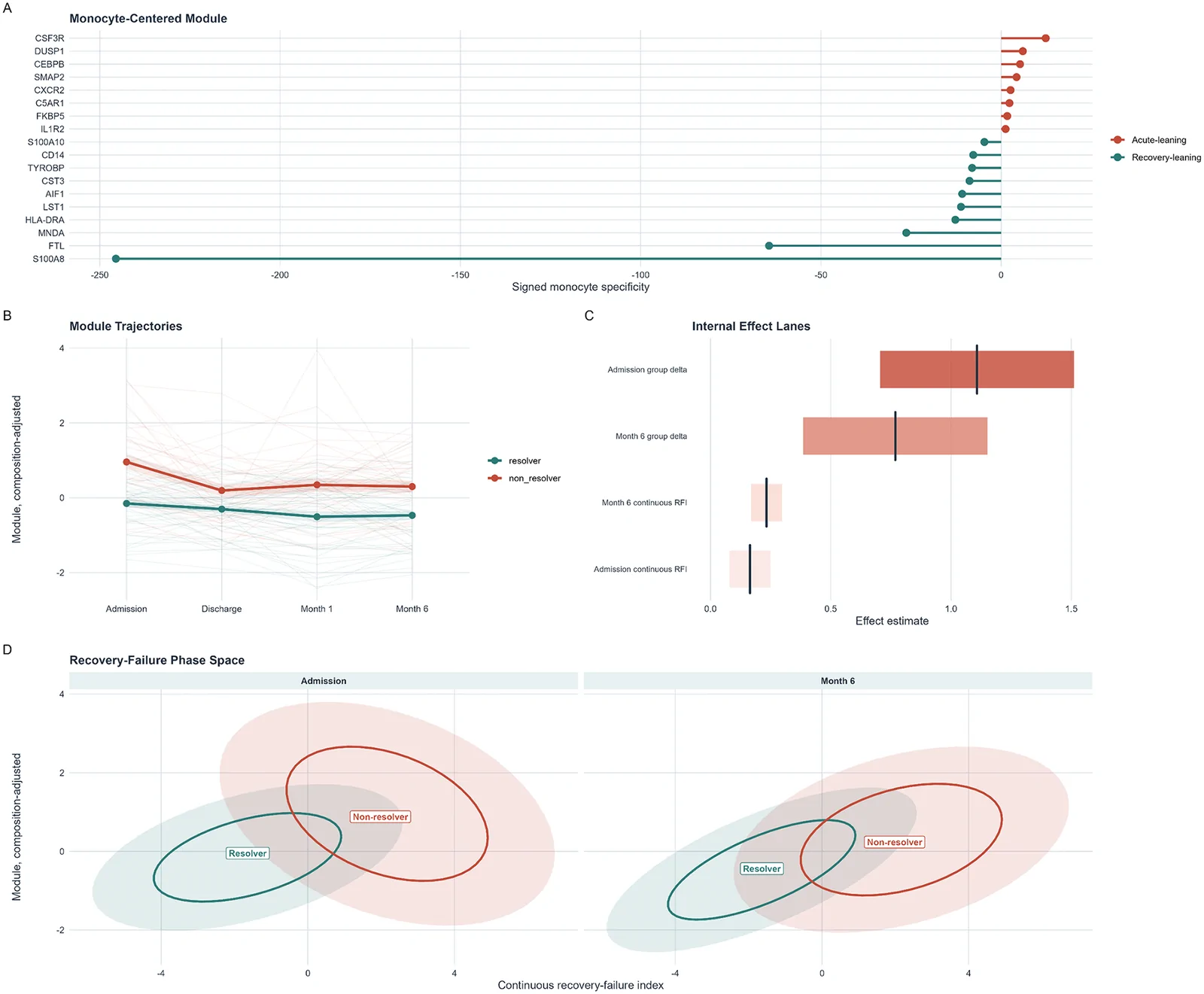
Construction of the recovery-failure module and support in the discovery cohort. A, Overview of monocyte specificity and directionality of the final module genes. B, Longitudinal module trajectories in GSE59867. C, Interval effect-band plot comparing admission and 6 months in the discovery cohort. D, Recovery-failure phase space defined by the continuous recovery-failure index and composition-adjusted module score.

Notably, the module was not constructed to maximize classification accuracy. It prioritized monocyte specificity and cross-layer consistency, and is therefore best interpreted as a biologically informed state module rather than a diagnostic or prognostic classifier.

3. The Recovery-Failure Module Shows External Temporal Transfer and Baseline-to-Follow-up Associations

To evaluate temporal transfer, we first tested the module in GSE62646, an external paired PBMC cohort. In STEMI patients, the module was directionally lower at 6 months than at admission, with borderline statistical evidence (β = −0.403, 95% CI −0.805 to −0.0001, P = 0.0499), consistent with a recovery-direction signal. In the larger whole-blood cohort GSE123342, the module was elevated in acute myocardial infarction at day 0 relative to coronary artery disease controls (β = 1.045, P = 2.05e-04), declined from day 0 to day 30 (β = −0.981, P = 8.86e-12), and remained lower than the acute phase at 1 year (β = −0.850, P = 7.69e-05). These findings support temporal transfer across sample types, platforms, and time windows, but they do not by themselves prove recovery-failure status in external cohorts lacking the same RFI definition (Fig 3).

**Fig 3:**
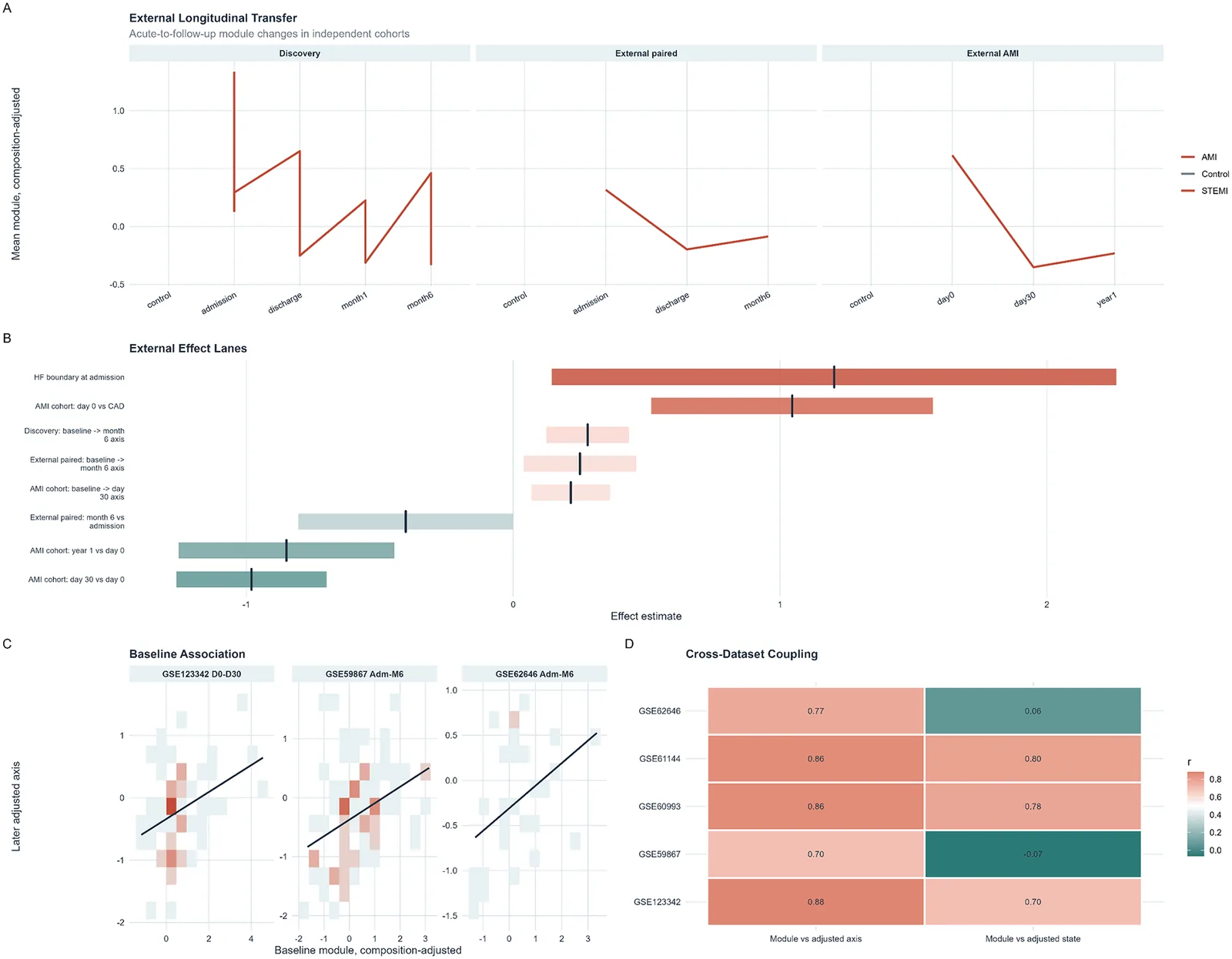
External temporal transfer and baseline-to-follow-up association. A, Temporal changes in mean module burden across GSE59867, GSE62646, and GSE123342. B, Interval effect-band plot for external temporal contrasts and baseline-to-follow-up association models. C, Binned-density plot of later composition-adjusted axis by baseline module burden. D, Heatmap of coupling between the module, composition-adjusted axis, and monocyte-state score across cohorts.

Baseline module burden was also associated with later residual inflammatory burden. In GSE59867, the admission module score was associated with the 6-month composition-adjusted axis (β = 0.279, P = 7.11e-04). In GSE62646, the admission module was associated with the 6-month composition-adjusted axis (β = 0.250, P = 0.0285). In GSE123342, the day-0 module score was associated with the day-30 composition-adjusted axis (β = 0.216, P = 0.00557). The module remained strongly correlated with the composition-adjusted axis in GSE123342 (r = 0.882) and was moderately correlated with the composition-adjusted monocyte-state score (r = 0.701). Overall, these results support a transferable longitudinal inflammatory-state signal rather than discovery-set-specific technical noise.

4. Single-Cell Anchoring Shows That Lesion-Associated Module Shifts Are Most Concentrated Within Monocytes

GSE269269 included 10 patients and 93,325 quality-controlled peripheral-blood mononuclear cells, including 53,933 cells from the rupture group and 39,392 cells from the non-rupture group. By cell type, T cells were the most abundant (51,015 cells), followed by monocytes (25,212 cells), NK cells (14,368 cells), B cells (867 cells), and platelets (1,863 cells). If the proportion of globally high-scoring cells was considered alone, T cells contributed most to the high-scoring cell pool because of their large cell number. However, after within-cell-type standardization, the strongest lesion-associated shift was not observed in T cells but in monocytes. This suggests that the recovery-failure module is not asking “which cell type is more abundant”, but rather “whether a given cell type has entered a specific state”. (S4 Fig)

In the Fig 4C (Fig 4) donor-level comparison, each cell type was summarized by donor before testing (5 rupture versus 5 non-rupture donors). The monocyte donor-level effect size, defined as the rupture minus non-rupture mean within-cell-type standardized module score, was −0.949 (rupture mean −0.467, non-rupture mean 0.482; two-sided Wilcoxon rank-sum P = 0.0079). The Fig 4C model was therefore a donor-level nonparametric group comparison rather than a cell-level test. Other major cell types showed smaller donor-level effect sizes in the same direction and were treated as descriptive rather than FDR-controlled significant findings. Because the dataset included only 10 patients, this result should be interpreted as single-cell anchoring evidence rather than definitive population-level inference.

**Fig 4:**
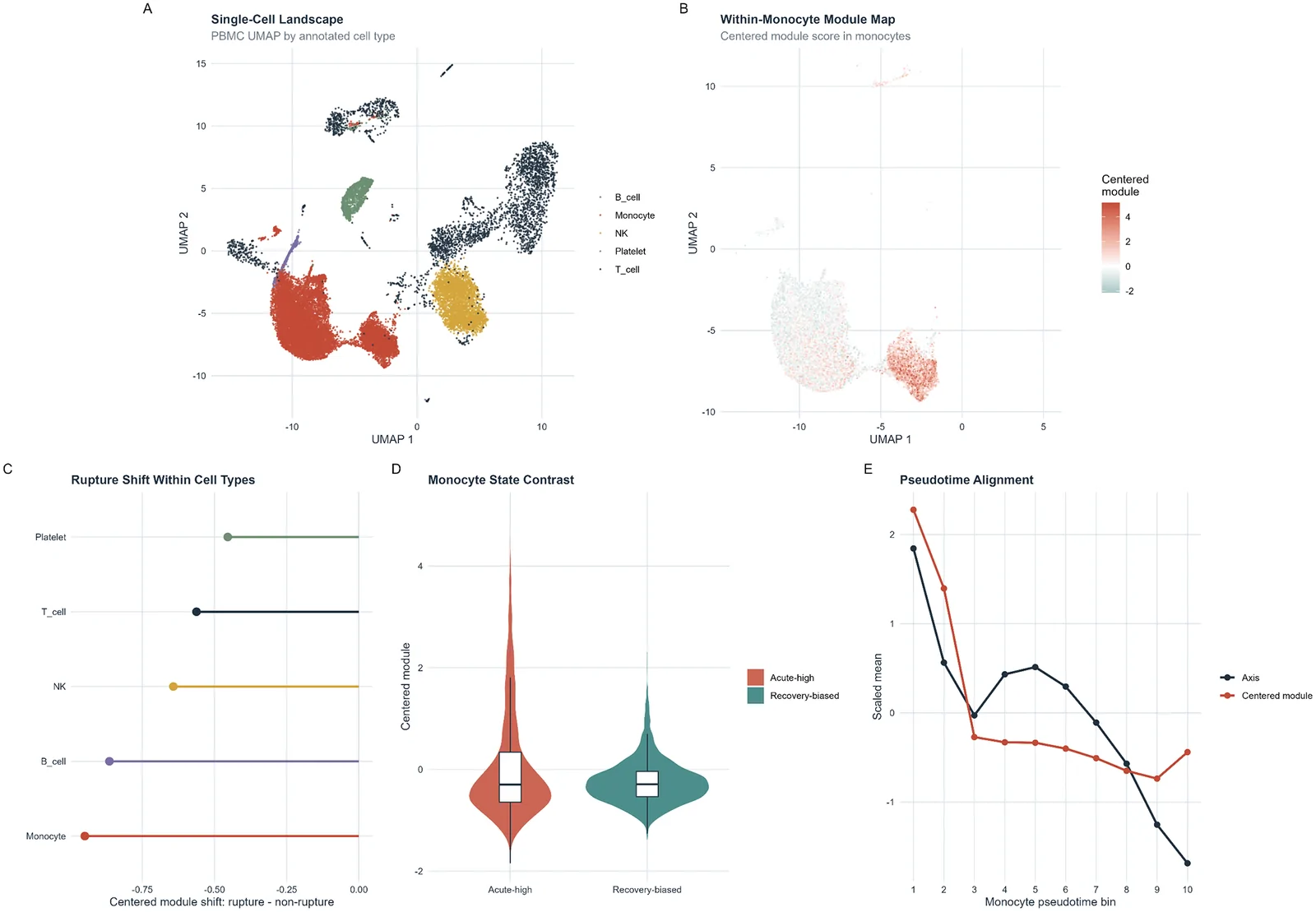
Single-cell anchoring. A, UMAP embedding of all cells in GSE269269. B, Distribution of the within-cell-type standardized module among monocytes in UMAP space. C, Donor-level within-cell-type standardized module differences between rupture and non-rupture lesions across cell types (effect size = rupture minus non-rupture mean score; two-sided Wilcoxon rank-sum tests on 5 rupture and 5 non-rupture donors). D, Distribution of within-cell-type standardized module scores in acute-high and recovery-biased monocytes. E, Joint changes in the module and original immune axis along acute-high-to-recovery-biased monocyte pseudotime.

Further, acute-high monocytes had a mean within-cell-type standardized module score of 0.029, whereas recovery-biased monocytes had a mean of −0.245, showing state-level separation. At the single-cell descriptive level, the module remained positively correlated with the original acute-to-recovery axis within monocytes (ρ = 0.460), indicating that the recovery-failure module remained aligned with the initial longitudinal axis definition while compressing it into a monocyte-associated state score.

5. Monocyte State Polarization, Communication Remodeling, and Pseudotime Provide Observational Mechanistic Support

We next compared acute-high and recovery-biased monocyte states defined by the original acute-to-recovery axis. Pseudobulk differential expression showed enrichment of inflammatory and myeloid activation pathways in acute-high monocytes, whereas recovery-biased monocytes showed relatively lower module burden and distinct antigen-presentation/remodeling-associated patterns. Together, these findings support the interpretation that the recovery-failure module is embedded in a monocyte-state differentiation structure, while remaining observational and hypothesis-generating.

At the ligand-receptor communication level, acute-high monocytes showed more prominent communication remodeling with lymphocytes and platelets than recovery-biased monocytes. For example, S100A8_TLR4, S100A9_TLR4, TIMP2_ITGB1, TYROBP_CD44, and VCAN-related interactions changed in directionally consistent ways in some recipient populations (Fig 5B). These interaction scores are computational summaries rather than functional assays, and they are presented as candidate downstream routes for future validation.

**Fig 5:**
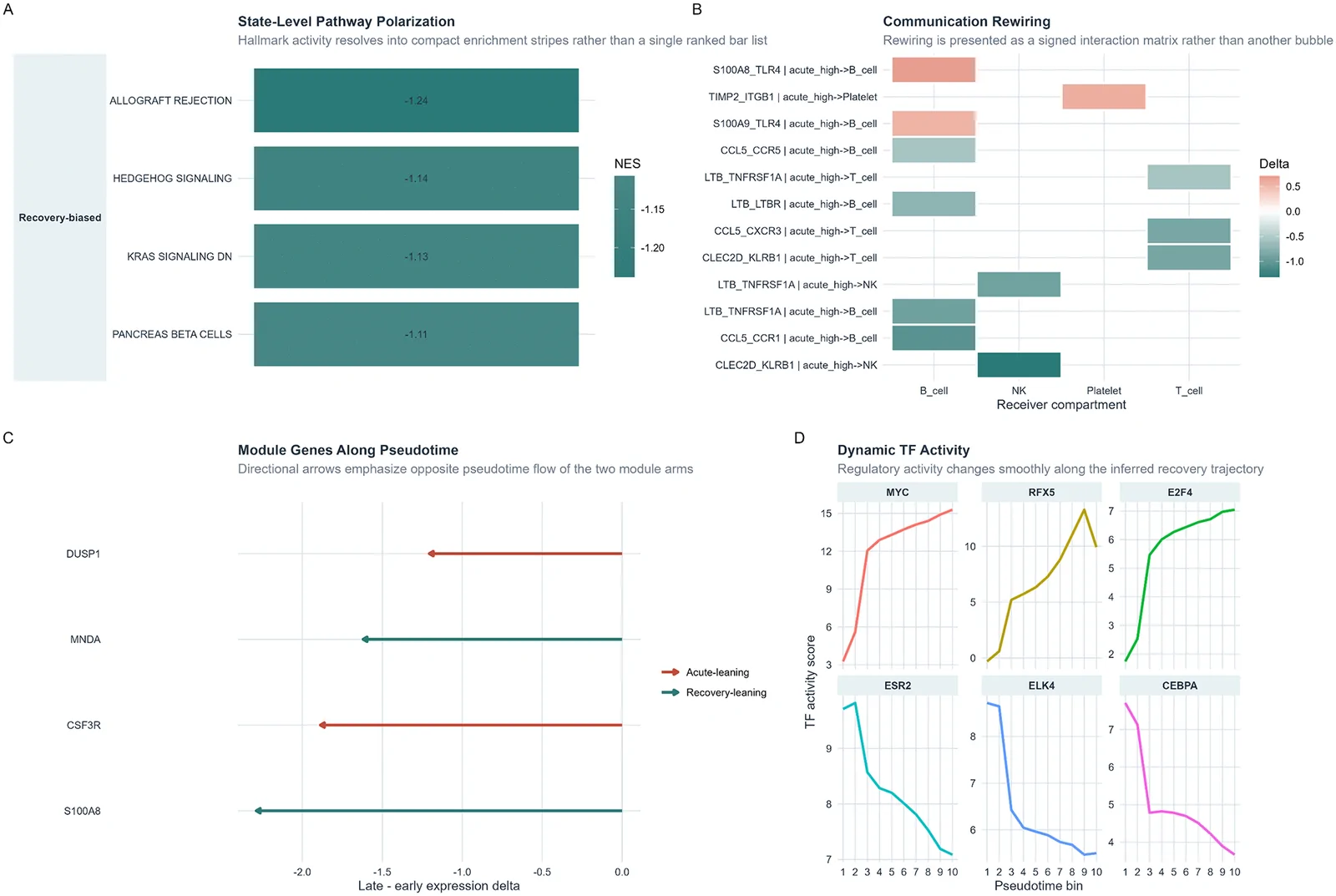
Observational monocyte-state support. A, Hallmark pathway enrichment strips for acute-high and recovery-biased monocytes. B, Ligand-receptor remodeling matrix from monocytes to lymphocytes and platelets. C, Directional arrow distribution of module genes along acute-high-to-recovery-biased pseudotime. D, Continuous trajectories of representative transcription factor activity along pseudotime.

After orienting pseudotime from acute-high toward recovery-biased monocytes, the recovery-failure module declined along the trajectory (Spearman ρ = −0.511). The positive gene arm was negatively correlated with pseudotime (ρ = −0.436), whereas the negative gene arm was positively correlated with pseudotime (ρ = 0.403). This suggests that the module combines two oppositely directed arms during the acute-to-recovery transition. Transcription-factor activity changed gradually across pseudotime bins (S5 Fig), supporting a continuous state-transition interpretation while not establishing causality (Fig 5C-D).

6. Robustness Comparisons Benchmark the Module Against Related Metrics

Finally, we compared the recovery-failure module with three related but distinct metrics: the composition-adjusted acute-to-recovery axis, monocyte-state transfer score, and monocyte abundance. In the temporal recovery comparison, monocyte abundance was most sensitive to raw time differences, as expected given marked myeloid mobilization in the acute phase. However, temporal separation is not equivalent to recovery-failure specificity. The recovery-failure module showed directionally consistent decline across four time contrasts, with a mean recovery statistic of 3.549. This was slightly lower than the composition-adjusted axis (3.833) but higher than the monocyte-state score (2.056) (Fig 6).

**Fig 6:**
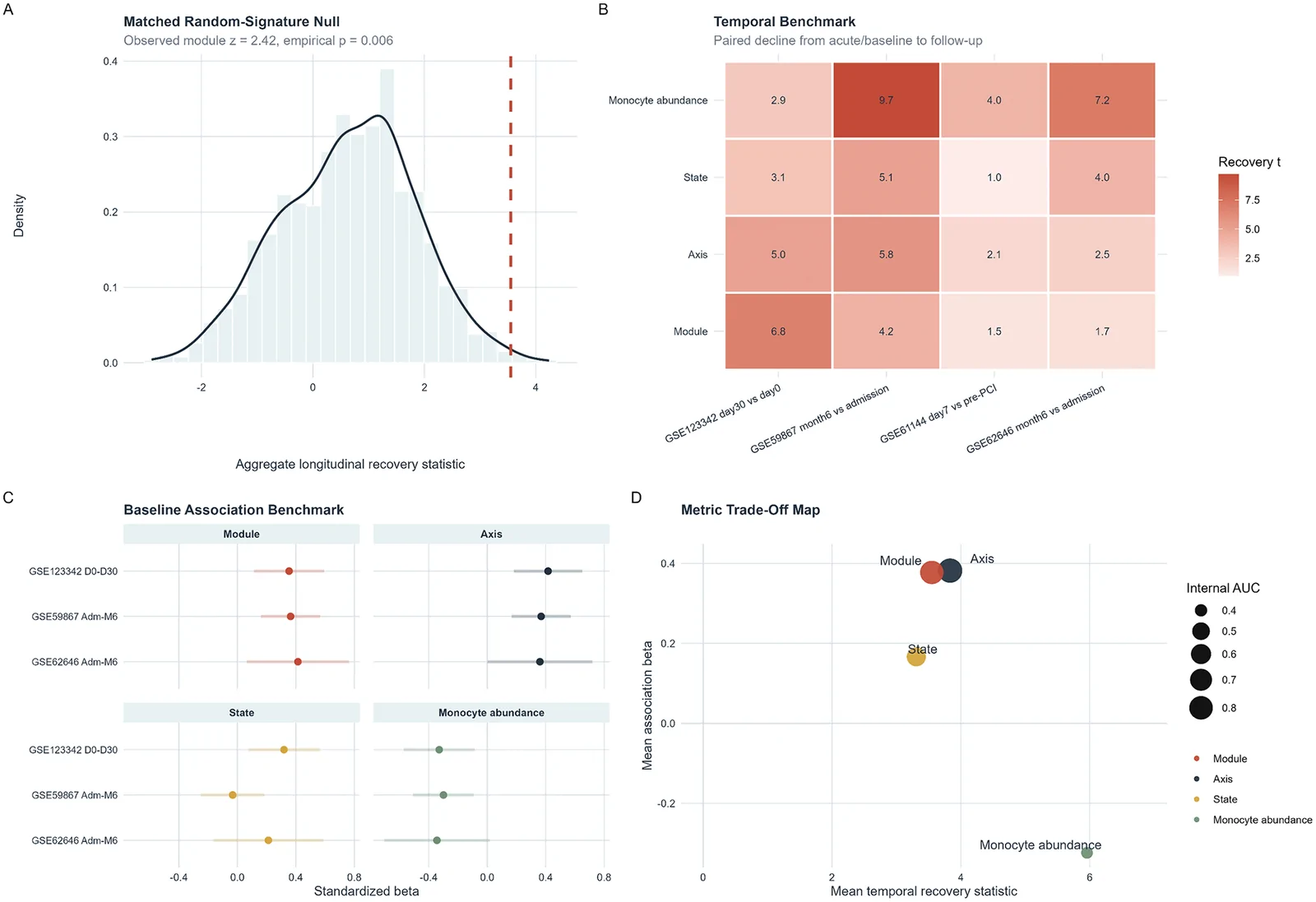
Robustness comparison. A, Random-control distribution from 1,000 matched random monocyte-centered gene sets. B, Heatmap comparing temporal recovery statistics. C, Faceted baseline-to-follow-up association bands comparing metrics. D, Trade-off map among temporal recovery ability, baseline-to-follow-up association, and internal classification performance.

In the baseline-to-follow-up association benchmark, the composition-adjusted axis had a mean standardized β of 0.382 and the recovery-failure module had a closely comparable mean standardized β of 0.377, both exceeding the monocyte-state score (0.166) and monocyte abundance (−0.324). In the internal classification comparison, the composition-adjusted axis had the highest area under the curve (0.853) because the recovery-failure phenotype was itself partly derived from the acute-to-recovery axis; the module had an area under the curve of 0.790. We therefore interpret this as an expected internal-definition advantage for the axis, not as a fair independent head-to-head classifier comparison.

In 1,000 matched random tests of candidate monocyte-centered gene sets, the observed module had a cross-cohort aggregate recovery statistic of 3.549, corresponding to a random-control Z value of 2.424 and an empirical P value of 0.006 calculated with a one-count continuity correction. Thus, when the comparison was restricted to monocyte-related candidate pools and preserved positive/negative arm sizes, the observed module exceeded 99.5% of random combinations. This supports non-random assembly of the module, while not replacing the need for external biological validation (Fig 6A).

7. Hard-Endpoint Support Remains Limited and Should Be Treated as Boundary Evidence Rather Than a Main Conclusion

We therefore retain hard-endpoint evidence as supplementary boundary results (S6 Fig) and emphasize that the strongest conclusion of this study is the existence, temporal transfer, and donor-aware single-cell anchoring of a dynamic immune recovery-failure program, rather than clinical event prediction itself.

## Discussion

The central contribution of this study is not the construction of another diagnostic signature for ACS, but the proposal of a reproducible dynamic immune framework for STEMI that follows the transition from acute inflammation toward recovery. Unlike most previous studies focusing on acute-phase cross-sectional comparisons, we first defined a recovery-failure phenotype in paired peripheral blood based on incomplete clearance of transcriptomic burden, then compressed it into a monocyte-associated molecular module, and finally evaluated its single-cell state alignment in a public AMI PBMC dataset. In other words, we built a state-axis framework rather than a diagnostic classifier.

This framework has three features worth emphasizing. First, it has longitudinal directionality. In the discovery cohort GSE59867, the external paired cohort GSE62646, and the large-sample time-series cohort GSE123342, the recovery-failure module was higher in the acute phase and declined during recovery, and baseline levels were associated with subsequent composition-adjusted axis burden. Second, it has donor-aware cellular anchoring. Through within-cell-type standardization in GSE269269, the strongest lesion-associated shift in donor-level summaries was observed within monocytes rather than in the globally most numerous high-scoring cell population. Third, it provides observational mechanistic support. Monocyte state polarization, communication remodeling, and pseudotime decline point to a monocyte-dominated recovery-impairment pattern, but they should be interpreted as hypothesis-generating because perturbational or functional validation was not performed.

From a pathobiological perspective, our findings are highly consistent with previous studies of post-myocardial-infarction inflammatory clearance, staged monocyte recruitment, and risk of adverse remodeling [10–19]. The difference is that we did not assume that a classic inflammatory molecule must be the answer. Instead, we allowed the public paired data to define “who did not recover” and then used single-cell data to answer which cells carry this state, how the state changes along cellular trajectories, and whether it is accompanied by communication remodeling. This “model first, anchor later” strategy may be better suited to public-data integration studies than starting from a single known pathway.

This study also addresses a common concern: bulk peripheral-blood signatures often act merely as surrogates for cell proportions. We observed that monocyte abundance was most sensitive to simple temporal separation. However, baseline-to-follow-up associations were not explained by abundance alone, and the single-cell lesion-associated shift remained largest in monocytes after within-cell-type standardization. These findings suggest that the module reflects monocyte state variation superimposed on cell-composition background, while acknowledging that single-cell inference was limited by the number of donors.

Several limitations should be acknowledged. First, all conclusions were derived from public retrospective datasets, and platforms, sample types, and follow-up time points were not fully consistent. Although this heterogeneity supported temporal transfer testing, it limited precise estimation of some effect sizes. Second, both the RFI and the binary recovery-failure phenotype were defined in GSE59867 from longitudinal transcriptomic structure rather than directly labeled by echocardiography, magnetic resonance imaging, or clinical endpoints; therefore, internal associations involving these quantities are vulnerable to circularity and require cautious interpretation. Third, external cohorts primarily demonstrate temporal decline and baseline-to-follow-up inflammatory association, not independently labeled recovery failure. Fourth, hard-endpoint evidence was weak, and the results for heart failure and recurrent events are insufficient to support clinical event-prediction claims. Fifth, GSE269269 included only 10 patients, so single-cell group comparisons were analyzed at the donor level and should be viewed as anchoring evidence rather than definitive population-level inference.

Therefore, the current work is best positioned as a purely bioinformatic transcriptomic integration study with observational mechanistic support. Future incorporation of large cohorts with left ventricular remodeling, heart-failure hospitalization, or imaging endpoints, together with quantitative PCR, flow-cytometry, and perturbational validation in independent samples, would substantially strengthen the clinical and biological persuasiveness of this framework.

## Conclusions

Using public peripheral-blood transcriptomic and single-cell data, we constructed a monocyte-centered STEMI immune recovery-failure framework. The framework distinguished transcriptomically defined recovery-sufficient and recovery-failure patients in the discovery cohort, showed temporal transfer across independent longitudinal cohorts, and localized in a public single-cell AMI context to donor-level monocyte state shifts and acute-to-recovery pseudotime changes. It is best positioned as a reproducible framework for dynamic immune stratification and hypothesis generation in ACS/STEMI rather than as a direct hard-endpoint prediction tool.

## Supporting information

S1 Fig Cohort design and quality-control overview. Summary of public cohort structure, score distributions, bulk preprocessing checks, and single-cell composition.
(TIFF)

S2 Fig Axis construction and composition adjustment. Source contrasts, gene-set behavior, cell-type marker construction, and composition-adjusted axis models.
(TIFF)

S3 Fig Module architecture and carrier analysis. Recovery-failure module gene evidence, score distributions, and cell-type carrier summaries.
(TIFF)

S4 Fig Single-cell state atlas. Monocyte state definitions, module distributions, and donor-level summaries in GSE269269.
(TIFF)

S5 Fig Communication, regulon, and random-module support. Ligand-receptor summaries, transcription-factor activity trends, and matched random-module comparisons.
(TIFF)

S6 Fig Exploratory endpoint analyses. Internal classification, GSE59867 heart-failure association, and GSE48060 recurrent-event analyses are presented as exploratory boundary evidence rather than main validation.
(TIFF)

## References

[1] Rao SV, O’Donoghue ML, Ruel M, Rab T, Tamis-Holland JE, Alexander JH, et al. 2025 ACC/AHA/ACEP/NAEMSP/SCAI guideline for the management of patients with acute coronary syndromes: A report of the american college of cardiology/american heart association joint committee on clinical practice guidelines. Circulation. 2025;151(13):e771–862. doi: 10.1161/CIR.0000000000001309 40014670

[2] Byrne RA, Rossello X, Coughlan JJ, Barbato E, Berry C, Chieffo A, et al. 2023 ESC Guidelines for the management of acute coronary syndromes. Eur Heart J. 2023;44(38):3720–826. doi: 10.1093/eurheartj/ehad191 37622654

[3] Thygesen K, Alpert JS, Jaffe AS, Chaitman BR, Bax JJ, Morrow DA, et al. Fourth universal definition of myocardial infarction (2018). Circulation. 2018;138(20):e618–51. doi: 10.1161/CIR.0000000000000617 30571511

[4] Libby P, Pasterkamp G, Crea F, Jang I-K. Reassessing the mechanisms of acute coronary syndromes. Circ Res. 2019;124(1):150–60. doi: 10.1161/CIRCRESAHA.118.311098 30605419 PMC6447371

[5] Virmani R, Burke AP, Farb A, Kolodgie FD. Pathology of the vulnerable plaque. J Am Coll Cardiol. 2006;47(8 Suppl):C13-8. doi: 10.1016/j.jacc.2005.10.065 16631505

[6] Bentzon JF, Otsuka F, Virmani R, Falk E. Mechanisms of plaque formation and rupture. Circ Res. 2014;114(12):1852–66. doi: 10.1161/CIRCRESAHA.114.302721 24902970

[7] Hansson GK, Hermansson A. The immune system in atherosclerosis. Nat Immunol. 2011;12(3):204–12. doi: 10.1038/ni.2001 21321594

[8] Libby P, Buring JE, Badimon L. Atherosclerosis. Nat Rev Dis Primers. 2019;5:56. 10.1038/s41572-019-0106-z 31420554

[9] Soehnlein O, Libby P. Targeting inflammation in atherosclerosis - from experimental insights to the clinic. Nat Rev Drug Discov. 2021;20(8):589–610. doi: 10.1038/s41573-021-00198-1 33976384 PMC8112476

[10] Ridker PM, Everett BM, Thuren T, MacFadyen JG, Chang WH, Ballantyne C, et al. Antiinflammatory therapy with canakinumab for atherosclerotic disease. N Engl J Med. 2017;377(12):1119–31. doi: 10.1056/NEJMoa1707914 28845751

[11] Tardif J-C, Kouz S, Waters DD, Bertrand OF, Diaz R, Maggioni AP, et al. Efficacy and safety of low-dose colchicine after myocardial infarction. N Engl J Med. 2019;381(26):2497–505. doi: 10.1056/NEJMoa1912388 31733140

[12] Frangogiannis NG. The inflammatory response in myocardial injury, repair, and remodelling. Nat Rev Cardiol. 2014;11(5):255–65. doi: 10.1038/nrcardio.2014.28 24663091 PMC4407144

[13] Frangogiannis NG. Regulation of the inflammatory response in cardiac repair. Circ Res. 2012;110(1):159–73. doi: 10.1161/CIRCRESAHA.111.243162 22223212 PMC3690135

[14] Swirski FK, Nahrendorf M. Cardioimmunology: The immune system in cardiac homeostasis and disease. Nat Rev Immunol. 2018;18(12):733–44. doi: 10.1038/s41577-018-0065-8 30228378

[15] Dutta P, Nahrendorf M. Monocytes in myocardial infarction. Arterioscler Thromb Vasc Biol. 2015;35(5):1066–70. doi: 10.1161/ATVBAHA.114.304652 25792449 PMC4409536

[16] Ma Y, Mouton AJ, Lindsey ML. Cardiac macrophage biology in the steady-state heart, the aging heart, and following myocardial infarction. Transl Res. 2018;191:15–28. doi: 10.1016/j.trsl.2017.10.001 29106912 PMC5846093

[17] Westman PC, Lipinski MJ, Luger D, et al. Inflammation as a driver of adverse left ventricular remodeling after acute myocardial infarction. J Am Coll Cardiol. 2016;67:2050–60. 10.1016/j.jacc.2016.01.073 27126533

[18] Tsujioka H, Imanishi T, Ikejima H, Kuroi A, Takarada S, Tanimoto T, et al. Impact of heterogeneity of human peripheral blood monocyte subsets on myocardial salvage in patients with primary acute myocardial infarction. J Am Coll Cardiol. 2009;54(2):130–8. doi: 10.1016/j.jacc.2009.04.021 19573729

[19] King KR, Aguirre AD, Ye Y-X, Sun Y, Roh JD, Ng RP Jr, et al. IRF3 and type I interferons fuel a fatal response to myocardial infarction. Nat Med. 2017;23(12):1481–7. doi: 10.1038/nm.4428 29106401 PMC6477926

[20] Vanhaverbeke M, Vausort M, Veltman D, Zhang L, Wu M, Laenen G, et al. Peripheral blood RNA levels of QSOX1 and PLBD1 are new independent predictors of left ventricular dysfunction after acute myocardial infarction. Circ Genom Precis Med. 2019;12(12):e002656. doi: 10.1161/CIRCGEN.119.002656 31756302 PMC6922070

[21] Suresh R, Li X, Chiriac A, Goel K, Terzic A, Perez-Terzic C, et al. Transcriptome from circulating cells suggests dysregulated pathways associated with long-term recurrent events following first-time myocardial infarction. J Mol Cell Cardiol. 2014;74:13–21. doi: 10.1016/j.yjmcc.2014.04.017 24801707 PMC4115027

[22] Wang Y, Chen Y, Zhang T. Integrated whole-genome gene expression analysis reveals an atlas of dynamic immune landscapes after myocardial infarction. Front Cardiovasc Med. 2023;10:1087721. doi: 10.3389/fcvm.2023.1087721 36937942 PMC10020602

[23] Fernandez DM, Rahman AH, Fernandez NF, Chudnovskiy A, Amir E-AD, Amadori L, et al. Single-cell immune landscape of human atherosclerotic plaques. Nat Med. 2019;25(10):1576–88. doi: 10.1038/s41591-019-0590-4 31591603 PMC7318784

[24] Depuydt MAC, Prange KHM, Slenders L, Örd T, Elbersen D, Boltjes A, et al. Microanatomy of the human atherosclerotic plaque by single-cell transcriptomics. Circ Res. 2020;127(11):1437–55. doi: 10.1161/CIRCRESAHA.120.316770 32981416 PMC7641189

[25] Mosquera JV, Auguste G, Wong D, Turner AW, Hodonsky CJ, Alvarez-Yela AC, et al. Integrative single-cell meta-analysis reveals disease-relevant vascular cell states and markers in human atherosclerosis. Cell Rep. 2023;42(11):113380. doi: 10.1016/j.celrep.2023.113380 37950869 PMC12335892

[26] Emoto T, Yamamoto H, Yamashita T, Takaya T, Sawada T, Takeda S, et al. Single-cell RNA sequencing reveals a distinct immune landscape of myeloid cells in coronary culprit plaques causing acute coronary syndrome. Circulation. 2022;145(18):1434–6. doi: 10.1161/CIRCULATIONAHA.121.058414 35500048

[27] Maciejak A, Kiliszek M, Michalak M, Tulacz D, Opolski G, Matlak K, et al. Gene expression profiling reveals potential prognostic biomarkers associated with the progression of heart failure. Genome Med. 2015;7(1):26. doi: 10.1186/s13073-015-0149-z 25984239 PMC4432772

[28] Park H-J, Noh JH, Eun JW, Koh Y-S, Seo SM, Park WS, et al. Assessment and diagnostic relevance of novel serum biomarkers for early decision of ST-elevation myocardial infarction. Oncotarget. 2015;6(15):12970–83. doi: 10.18632/oncotarget.4001 26025919 PMC4536992

[29] Kiliszek M, Burzynska B, Michalak M, Gora M, Winkler A, Maciejak A, et al. Altered gene expression pattern in peripheral blood mononuclear cells in patients with acute myocardial infarction. PLoS One. 2012;7(11):e50054. doi: 10.1371/journal.pone.0050054 23185530 PMC3503717

[30] Veltman D, Wu M, Pokreisz P, Claus P, Gillijns H, Caluwé E, et al. Clec4e-receptor signaling in myocardial repair after ischemia-reperfusion injury. JACC Basic Transl Sci. 2021;6(8):631–46. doi: 10.1016/j.jacbts.2021.07.001 34466750 PMC8385568

[31] Qian J, Gao Y, Lai Y, Ye Z, Yao Y, Ding K, et al. Single-Cell RNA sequencing of peripheral blood mononuclear cells from acute myocardial infarction. Front Immunol. 2022;13:908815. doi: 10.3389/fimmu.2022.908815 35844519 PMC9278132

[32] Stuart T, Butler A, Hoffman P, Hafemeister C, Papalexi E, Mauck WM 3rd, et al. Comprehensive integration of single-cell data. Cell. 2019;177(7):1888-1902.e21. doi: 10.1016/j.cell.2019.05.031 31178118 PMC6687398

[33] Hao Y, Hao S, Andersen-Nissen E, Mauck WM 3rd, Zheng S, Butler A, et al. Integrated analysis of multimodal single-cell data. Cell. 2021;184(13):3573-3587.e29. doi: 10.1016/j.cell.2021.04.048 34062119 PMC8238499

[34] Korsunsky I, Millard N, Fan J, Slowikowski K, Zhang F, Wei K, et al. Fast, sensitive and accurate integration of single-cell data with Harmony. Nat Methods. 2019;16(12):1289–96. doi: 10.1038/s41592-019-0619-0 31740819 PMC6884693

[35] Barrett T, Wilhite SE, Ledoux P, Evangelista C, Kim IF, Tomashevsky M, et al. NCBI GEO: Archive for functional genomics data sets--update. Nucleic Acids Res. 2013;41(Database issue):D991-5. doi: 10.1093/nar/gks1193 23193258 PMC3531084

[36] Davis S, Meltzer PS. GEOquery: A bridge between the Gene Expression Omnibus (GEO) and BioConductor. Bioinformatics. 2007;23(14):1846–7. doi: 10.1093/bioinformatics/btm254 17496320

[37] Ritchie ME, Phipson B, Wu D, Hu Y, Law CW, Shi W, et al. limma powers differential expression analyses for RNA-sequencing and microarray studies. Nucleic Acids Res. 2015;43(7):e47. doi: 10.1093/nar/gkv007 25605792 PMC4402510

[38] Robinson MD, McCarthy DJ, Smyth GK. edgeR: A Bioconductor package for differential expression analysis of digital gene expression data. Bioinformatics. 2010;26(1):139–40. doi: 10.1093/bioinformatics/btp616 19910308 PMC2796818

[39] Liberzon A, Birger C, Thorvaldsdottir H, Ghandi M, Mesirov JP, Tamayo P. The Molecular Signatures Database Hallmark Gene Set Collection. Cell Syst. 2015;1:417–25. 10.1016/j.cels.2015.12.004 PMC4707969 26771021

[40] Subramanian A, Tamayo P, Mootha VK, Mukherjee S, Ebert BL, Gillette MA, et al. Gene set enrichment analysis: A knowledge-based approach for interpreting genome-wide expression profiles. Proc Natl Acad Sci U S A. 2005;102(43):15545–50. doi: 10.1073/pnas.0506580102 16199517 PMC1239896

[41] Türei D, Valdeolivas A, Gul L, Palacio-Escat N, Klein M, Ivanova O, et al. Integrated intra- and intercellular signaling knowledge for multicellular omics analysis. Mol Syst Biol. 2021;17(3):e9923. doi: 10.15252/msb.20209923 33749993 PMC7983032

[42] Street K, Risso D, Fletcher RB, Das D, Ngai J, Yosef N, et al. Slingshot: Cell lineage and pseudotime inference for single-cell transcriptomics. BMC Genomics. 2018;19(1):477. doi: 10.1186/s12864-018-4772-0 29914354 PMC6007078

[43] Garcia-Alonso L, Holland CH, Ibrahim MM, Turei D, Saez-Rodriguez J. Benchmark and integration of resources for the estimation of human transcription factor activities. Genome Res. 2019;29(8):1363–75. doi: 10.1101/gr.240663.118 31340985 PMC6673718

[44] Badia-I-Mompel P, Vélez Santiago J, Braunger J, Geiss C, Dimitrov D, Müller-Dott S, et al. decoupleR: ensemble of computational methods to infer biological activities from omics data. Bioinform Adv. 2022;2(1):vbac016. doi: 10.1093/bioadv/vbac016 36699385 PMC9710656

